# Supplementary material for: Mitochondrial ferredoxin-like is essential for forming complex I-containing supercomplexes in Arabidopsis
Source: Plant Physiol. 2023 Jan 25;191(4):2170–84. doi: 10.1093/plphys/kiad040 (PMC10069907; doi:10.1093/plphys/kiad040)
Supplement: kiad040_Supplementary_Data [file kiad040_supplementary_data.pdf]

```
                    10         20         30         40         50         60         70
            ....|....| ....|....| ....|....| ....|....| ....|....| ....|....| ....|....|
AtmFDX-like ---------- ---------- ---------- ----MATTLQ KLSSQIHR-- ---LSPFTRS LIVRTSATSA   31
AtmFDX1     MIGHRISRLG STIVKQLARE GYLATYGTKN LHRSYGHYLQ SLPVVPRQ-- ---ARTSQEA WFLKSH-KFC   64
AtmFDX2     MVFHRLSRLG SRIVKELPRE RHLSMCGKRI LQRSYGQYLQ SSPMLQRQ-- ---TRSFKEA LFSNNH-KFC   64
ScYah1      ---------- --MLKIVTRA GH-----TAR ISNIAAHLLR TSPSLLTR-- ---TTTTTRF LPFSTS-SFL   47
HsmFDX1     ---------- ----MAAAGG ARLLRAASAV LGGPAGRWLH HAGSRAGS-- ---SGLLRNR GPGGSA-EAS   50
HsmFDX2     ---------- --MAASMARG G---VSARVL LQAARGTWWN RPGGTSGSGE GVALGTTRKF QATGSR-PAG   54

                    80         90        100        110        120        130        140
                 ▼
            ....|....| ....|....| ....|....| ....|....| ....|....| ....|....| ....|....|
AtmFDX-like PSPSLGSKKV SDRI--VKLS AIDPDGYKQD IIGLSGQTLL RALTHTGLID PASHRLDDIE ACSAECEVQI   99
AtmFDX1     TSSTTSSENG DEETEKITII FVDKDGEEIP VKVPIGMSVL EAAHENDIDL EGA--CEASL ACST-CHVIV   131
AtmFDX2     TSFSTTSEKG GEKTEKINVT FVDKDGEEIH IKVPVGMNIL EAAHENDIEL EGA--CEGSL ACST-CHVIV   131
ScYah1      NHGHLKKPKP GEE---LKIT FILKDGSQKT YEVCEGETIL DIAQGHNLDM EGA--CGGSC ACST-CHVIV   111
HsmFDX1     RSLSVSARAR SSSEDKITVH FINRDGETLT TKGKVGDSLL DVVVENNLDI DGFGACEGTL ACST-CHLIF   119
HsmFDX2     EEDAGGPERP GDV---VNVV FVDRSGQRIP VSGRVGDNVL HLAQRHGVDL EGA--CEASL ACST-CHVYV   118
                                                                        *      *   *

                   150        160        170        180        190        200
            ....|....| ....|....| ....|....| ....|....| ....|....| ....|....| ....|....
AtmFDX-like -AEEWLEKLP PRTYDEEYVL KRSSRSRILN KHSRLGCQVV LTQELQGMVV AVPEA----- KPWDIP---   159
AtmFDX1     MDTEYYNKLE EPTDEENDML DLAFG---LT ETSRLGCQVI ARPELDGVRL AIPSATRNFA VDGFVPKPH   197
AtmFDX2     MDTKYYNKLE EPTDEENDML DLAFG---LT ATSRLGCQVI AKPELDGVRL AIPSATRNFA VDGFVPKPH   197
ScYah1      -DPDYYDALP EPEDDENDML DLAYG---LT ETSRLGCQIK MSKDIDGIRV ALPQMTRNVN NNDFS----   172
HsmFDX1     -EDHIYEKLD AITDEENDML DLAYG---LT DRSRLGCQIC LTKSMDNMTV RVPETVADAR QSIDVGKTS   184
HsmFDX2     -SEDHLDLLP PPEEREDDML DMAPL---LQ ENSRLGCQIV LTPELEGAEF TLPKITRNFY VDGHVPKPH   183
                          ● ● ●   ●                *
```

Supplemental Figure S1. Amino acid sequence alignment of AtmFDX-like and mitochondrial ferredoxin homologues.

Conserved cysteine residues required for the [2Fe-2S] cluster coordination are pointed by asterisks whereas acidic amino acid residues a priori involved in the interaction with the ferredoxin reductase based on the structure of the adrenodoxin/adrenodoxin reductase complex (Muller et al., 2001) are indicated by red rings. Chosen cleavage sites for recombinant protein production are represented by an orange triangle. *A. thaliana* (At), *S. cerevisiae* (Sc) and *H. sapiens* (Hs). Shaded areas indicate at least 80% amino acid identity (black) or similarity (grey). The UniProt accession numbers are: AtmFDX-like: Q9SRR8, AtMFDX1: Q9M0V0, AtmFDX2: Q8S904, ScYah1: Q12184, HsmFDX1: P10109, HsmFDX2: Q6P4F2.

MULLER, J. J., LAPKO, A., BOURENKOV, G., RUCKPAUL, K. & HEINEMANN, U. 2001. Adrenodoxin reductase-adrenodoxin complex structure suggests electron transfer path in steroid biosynthesis. *J Biol Chem,* 276**,** 2786-9.

```
                                     10        20        30        40        50        60
                             ....|....| ....|....| ....|....| ....|....| ....|....| ....|....|
Arabidopsis thaliana         -MATTLQKLS SQIH--RLSP FTRSL---IV RTSATS---- --APSPSLGS KKVSDRIVKL  48
Brassica rapa                -MATNLLKLS SQIR--RLSP LTRSL---TV RTSATS---- -------TGS KKVSDRIVKL  43
Capsella rubella             -MATTLQKLS SQIH--RLSP FVRSL---TV RTSATS---- --APSPSLGS KKVSDRIVKL  48
Thlaspi arvense              -MATHLQKLS SQIH--RLSP FARSV---II RTSATSAPVS -VAPSPSSGT RKVADRIVKL  53
Solanum lycopersicum         MAIAYLRRFA SRIA--RNTS LSSSFGSSIA RSSASS---- ----SSPSAS AKVADRIVKL  50
Selaginella moellendorffii   -MADLL---P VQVR------ ---------- ---------- ----QPGDSL PRVDTRIVTA  26
Physcomitrella patens        MAMAAAQSLR GSRR--HLLS YVRYGGSIAT QSESFV---- -----VRQGS AKVSDRVLEM  49
Sphagnum fallax              -MAMAMVAGR RCLQ--RAVR HAATAAAPAV QVTQES---- --TRREAGRS AKVSDRMLEM  51
Marchantia polymorpha        -MATALRRL- VQVR------ --------FA HTAVAS---- -VSSAIPRSA PKVLESTVRL  39
Volvox carteri               -MA--LRALG RQLRSLNLIP VVQPR--RFF GASAHHDDEH -EEEHGPAQT PTVFDKLVEI  54
Dunaliella salina            MLKAAGASLR RALAAVSTSP LESSS---RL IASSSAVQGG ADEHHGYEQT PTVFDRLVTI  57
Chlamydomonas reinhardtii    -MA--LRALG RQLRSLNLLP AVQPA--RFF GAGAHHDDEH EEEEHGPAQT PTVFDKLVEV  55
Polytomella sp.              -MA-LLRALA KPLRSLQAVS SVAQVSLRQF GAASHHDDHH -DDHDHYTPP KTVFEDTITI  57

                                     70        80        90       100       110       120
                             ....|....| ....|....| ....|....| ....|....| ....|....| ....|....|
Arabidopsis thaliana         SAI-DPDGYK QDIIGLSGQT LLRALTHTGL IDPASHRLDD IEACS---AE CEVQIAEEWL  104
Brassica rapa                SAI-DADGDK KDIIGLSGQT LLRALTHTGL IDPLSHRLED IDACS---AE CEVQIAEEWL  99
Capsella rubella             SAI-DPDGYK QDIIGLSGQT LLRALTHTGL IDPASHRLDD IEACS---AE CEVQIAEEWL  104
Thlaspi arvense              SAI-DADGYK KEIIGLSGQT LLRALTHTGL IDPASHRLDD IEACS---AE CEVQIAEEWL  109
Solanum lycopersicum         SAV-DPDGNK RDVVGLSGQT LLKALTNQGL IDPDSHRLED IDACS---AE CEVHIAQEWL  106
Selaginella moellendorffii   IAV-DEDGRR HTVRALAGQS LFRALVNAGL RLHLSHQL-D TSCYG---YV CRVSISDEWR  81
Physcomitrella patens        TAI-DEDGQR HQIKGLTGHT LLRTLVERGL FDPERHRLEN INACG---GE CEVSIANEWL  105
Sphagnum fallax              TAI-DEDGER HVVKGLTGHT LLRTLVQSGL FDPERHRLED ITACN---GE CEVSIANEWL  107
Marchantia polymorpha        TAIAEENGSR HAIRGLVGQT LLKTLVRSGL IEGESHRTED LTQCG---AE CEVSVAGEWL  96
Volvox carteri               TVV-DMNGVL HKVRGLAGQT LAQALIEYGF --PATYFFPN MGFYTQHIPD AHVYVPKDYW  111
Dunaliella salina            NVV-DLDGKR RSVRAVVGQT LAQVLVEAGY --PRTYFFPN MGFYTQHIVD AHVFIPQDYW  114
Chlamydomonas reinhardtii    TVV-DMNGIR HRVRGLQGQS LAQALVEYGF --PDTYFFPN MGFYTQHIVD AHVFVPKEFW  112
Polytomella sp.              NVL-DYDGKK HAVKALIGTP LNKALVEYGF --SSTYFFPN MGYYTQHISD AHVFIPEEYW  114
                                                                           *      *
                                    130       140       150       160       170       180
                             ....|....| ....|....| ....|....| ....|....| ....|....| ....|....|
Arabidopsis thaliana         EKLP--PRTY DEEYVL-KRS SRSRILN--- KHSRLGCQVV LTQELQGMVV AVPEAKPWDI  158
Brassica rapa                EKLP--PRTY DEEYVL-KRN SRSRVLN--- KHSRLGCQVV LTQELQGMVV AVPEAKPWDI  153
Capsella rubella             EKLP--PRSY DEEYVL-KRS SRSRVLN--- KHSRLGCQVV LTQELQGMVV AVPEAKPWDI  158
Thlaspi arvense              EKLP--TRTY DEEYVL-RRN SRSRVLN--- KHSRLGCQVV LTQELQGMVV AVPEAKPWDI  163
Solanum lycopersicum         EKLP--PASY DEKYVL-KRN SRARVLN--- KHSRLGCQVV LSQELQGMVV ALPEPKPWDI  160
Selaginella moellendorffii   ARIP--EPTE DELHVL-KHH FTPPDFD--- PSIRCSCKIK LSDKINGIVV ALVPEKCWDF  135
Physcomitrella patens        DKLP--PRSE DELEVL-KDK THAKKAD--- PHARLGCQIV LEPELEGMVV SIAEEKPWRT  159
Sphagnum fallax              VKLP--PRTE DELDVL-QAK TFSKLAD--- PHSRLGCQII LEPELEGMVV SIAEEKPWRT  161
Marchantia polymorpha        EKLP--PRSE DEMEILEKHQ PKGQAVD--- THTRLACQIV LDRKLDGMAF AIPEARPWMS  151
Volvox carteri               GKMPNIDPES EEGGAV-KRM FRDIVQDYQR DTSYFASYIT LGPELNGMTV GIGPIKPWIL  170
Dunaliella salina            SKIRNYKEDS DEADAI-KRT FRDMIQDYAK STSYLASYID IVPEFNNMTI GIGPVKPWVL  173
Chlamydomonas reinhardtii    GKVQNVDPES DDGLAV-KRM FRDIVQDYQR DTSFFASYIT LGAEHNGMTV GIGPIKPWIL  171
Polytomella sp.              KYVENVDLKT DDAEAI-KLM FKLVVQDYQR ETSFFASYLT LNKEMDNMTI GFGPIKPWHI  173
                                                                    *
                                    190       200       210
                             ....|....| ....|....| ....|....|
Arabidopsis thaliana         P--------- ---------- ----------  159
Brassica rapa                P--------- ---------- ----------  154
Capsella rubella             P--------- ---------- ----------  159
Thlaspi arvense              P--------- ---------- ----------  164
Solanum lycopersicum         P--------- ---------- ----------  161
Selaginella moellendorffii   T--------- ---------- ----------  136
Physcomitrella patens        L--------- ---------- ----------  160
Sphagnum fallax              L--------- ---------- ----------  162
Marchantia polymorpha        N--------- ---------- ----------  152
Volvox carteri               HSDWAFAGVH DSKAKQFDK- ---PTVEIWG  196
Dunaliella salina            HSEWAFNGVH DDPTPQFKE- ---PTTEIWG  199
Chlamydomonas reinhardtii    HSEWAFAGVH DSRNKQFDK- ---PTVEIWG  197
Polytomella sp.              TPKWSFNGHH NVKDRMFDRL ETGPFIE---  200
```

Supplemental Figure S2. Amino acid sequence alignment of selected plant and algal mFDX-like proteins.

The three remaining cysteines known to serve as [2Fe-2S] cluster ligands in regular FDXs are pointed by asterisks. Shaded areas indicate at least 80% amino acid identity (black) or similarity (grey). The UniProt accession numbers are: *Arabidopsis thaliana*: Q9SRR8, *Brassica rapa*: M4ELI3, *Capsella rubella*: R0I0R4, *Solanum lycopersicum*: A0A3Q7GPB6, *Selaginella moellendorffii*: D8RBK4, *Physcomitrella patens*: A0A2K1L3S2, *Marchantia polymorpha*: A0A2R6WIY0, *Volvox carteri*: D8TW15, *Chlamydomonas reinhardtii*: Q6QAY1. The sequences absent from UniProt were retrieved from the following sources: Phytozome: *Thlaspi arvense*: Thlar.0128s0036, *Sphagnum fallax*: Sphfalx07G032200, *Dunaliella salina*: Dusal.0767s00002 and NCBI: *Polytomella sp.*: 7AR9_O.

*

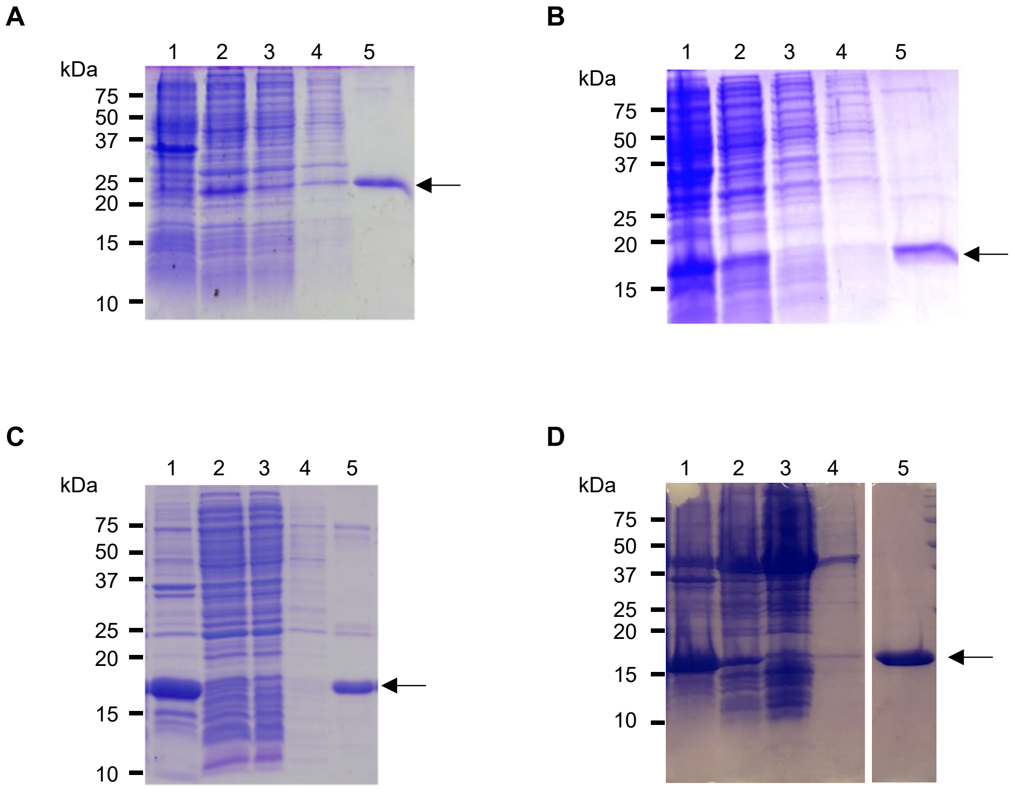

Supplemental Figure S3. Purification steps and purity degree of mrecombinant proteins.

Fractions obtained during aerobic protein purification of tagged proteins were analyzed on 15% SDS-PAGE. 1. insoluble fraction, 2. soluble fraction, 3. flowthrough fraction, 4. washing fraction and 5. elution fraction. A. mFDX1, B. mFDX2, C. mFDX-like, D. mFDX-like L85C. The arrows indicate the bands corresponding to the expected size of each protein. In D, the gel was cut because of an intermediate lane but it is the same gel.

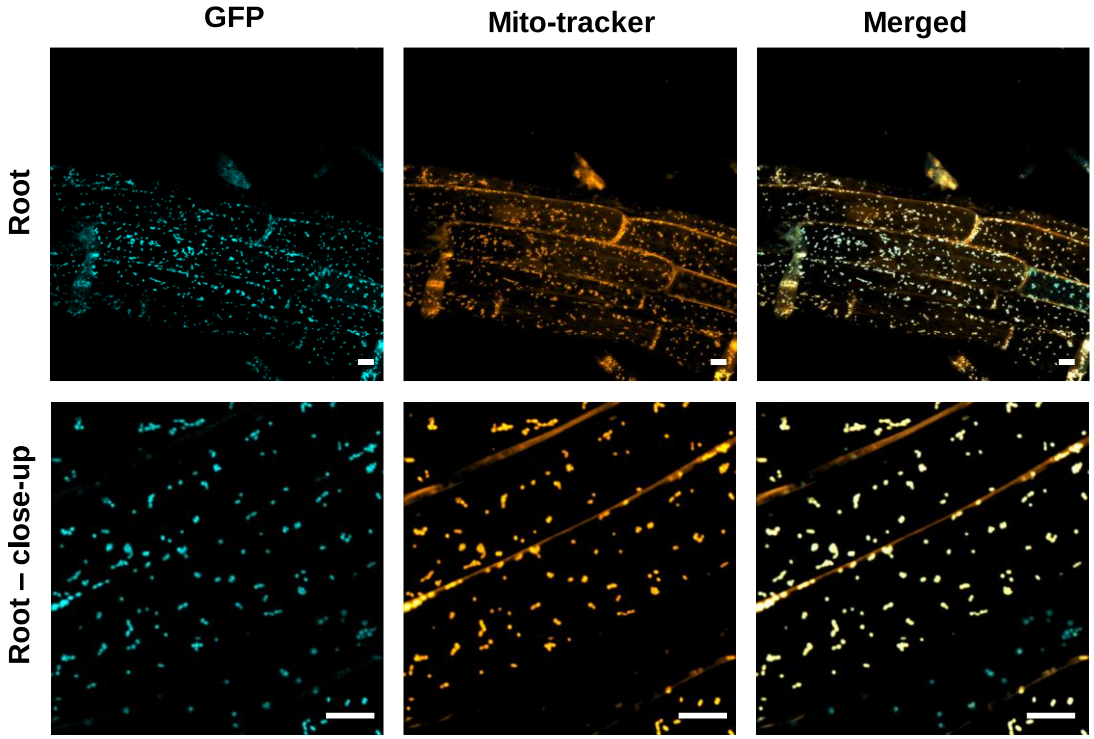

Supplemental Figure S4. Subcellular localization of AtmFDX-like in Arabidopsis. Expression of *promoter-mFDX-like::mFDX-like::GFP* in root cells from a 14-day-old seedling. GFP, cyan; MitoTracker, orange. Scale bar: 10 µm.

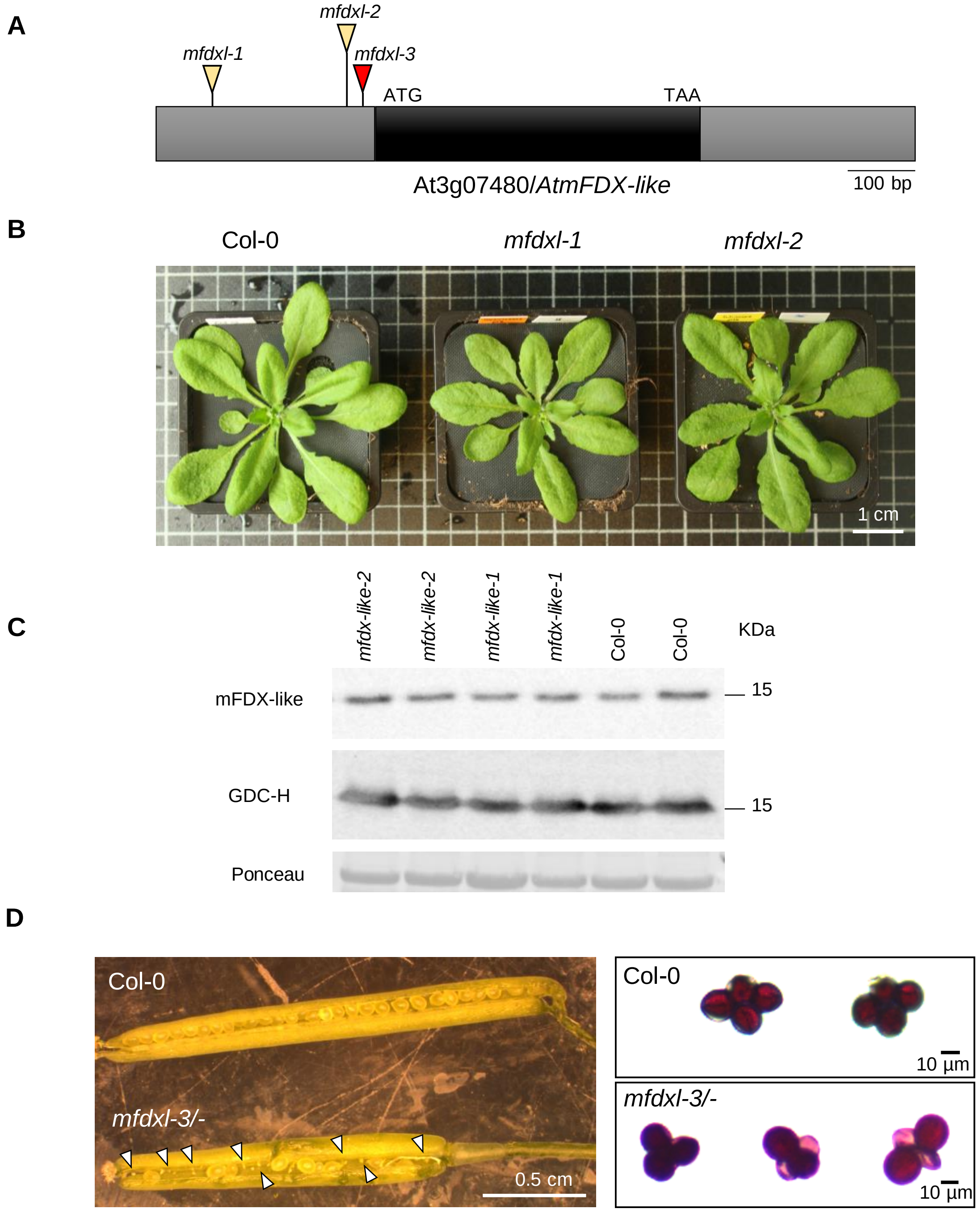

Supplemental Figure S5. Genetic analysis and characterization *mfdx-like* mutants from *Arabidopsis thaliana*.
(A) Gene structure of the *mFDX-like* gene, At3g07480. Exons are represented in black, both 5' and 3' untranslated (UTRs) regions are shown as grey rectangle. Positions of T-DNA insertions of the mutant lines are represented by triangles. *mfdxl-1* : SALK_015333; *mfdxl-2* : WiscDsLoxHs204_01A; *mfdxl-3*: SAIL_37_H11. (B) Growth phenotypes of 24 day-old wild-type Col-0 (WT), *mfdx-l-1* and *mfdx-l-2* plants grown under long-day conditions. (C) Abundance of mFDX-like protein levels determined by western-blot. These representative western-blots were performed from mitochondria-enriched extract isolated from leaves. 20 µg of soluble proteins was loaded per lines. Levels of H-proteins and Ponceau S stain was used to confirm equal loading and transfer. (D) Siliques from a wild-type (top) and *mfdxl-3/-* (bottom) were opened and immature seeds are shown. Mature pollen grains were collected from wild-type and heterozygous *mfdxl-3* plants, and viability was tested by using Alexander's stain. Cytoplasm of viable pollen grains was coloured in purple whereas dead cells appeared transparent and shrivelled. For each genotype, all images were taken at the same magnification.

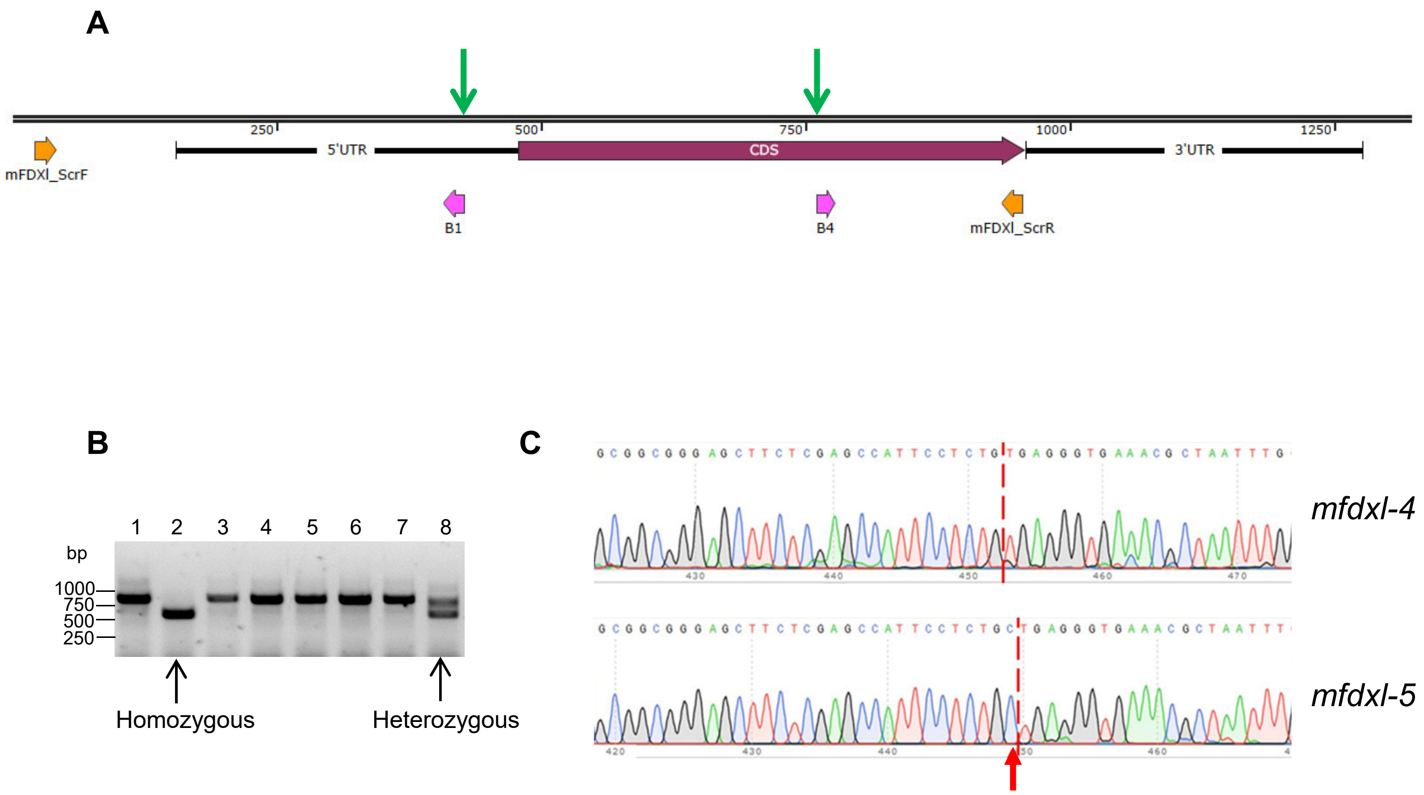

Supplemental Figure S6. CRISPR/Cas9 strategy to obtain deletion mutants

A. Schematic representation of the gene *At3g07480* coding for mFDX-like. The protospacer sequences of the two guide RNAs (B1 and B4) used to create the deletion are indicated with pink arrows. The expected Cas9 cut sites are indicated with green arrows. The sequence between the two cut is lost after repair of the DNA. The position of the primers used to screen the mutant lines is indicated with orange arrows. Numbers indicate the number of nucleotides from the left end of the scheme. B. Eight T2 plants were screened by PCR using the primers mFDXl_ScrF and mFDXl-ScrR. The WT PCR product has a size of 934 bp, the deletion product has an expected size of ca. 600 bp. No signal is detected at 934 bp in plant 2, this plant is thus considered homozygous. C. Sequence chromatograms obtained by sequencing the 600 bp PCR product using the primer mFDXl_ScrR. The mutant *mfdxl-4* shows the expected repair site (indicated with a dashed red line) whereas the mutant *mfdxl-5* contains an additional C (indicated with a red arrow) at the repair site (dashed red line).

Supplemental Table S1: **UniProt accession numbers of the proteins used for the phylogenetic study.**

Eukaryotic groups are based on (Keeling and Burki 2019). For each organism, the different FDXs were identified using PSI-BLAST using the Arabidopsis mFDXs as baits. The cor espon ing UniProt accession given. The chloroplastic FDXs are indicated in green

Group	Class	Organism	Name	Uniprot
Amoebozoa	Discosea	*Acanthamoeba castellanii*	AcFDX	L8GR90
	Dictyostelia	*Dictyostelium discoideum*	DdFDX	Q55GW1
	Apusomonadea	*Thecamonas trahens*	ThFDX	A0A0L0DMW7
Opisthokonta	Chordata	*Homo sapiens*	HsFDX1	P10109
			HsFDX2	Q6P4F2
	Ascomycota	*Yarrowia lipolytica*	YlFDX	Q6CFZ5
Archaeplastida	Tracheophyta	*Arabidopsis thaliana*	AtcFDX1	O04090
			AtcFDX2	P16972
			AtcFDX3	Q9ZQG8
			AtcFDX4	Q9FIA7
			AtcFDXC1	O23344
			AtcFDXC2	Q9C7Y4
			AtmFDX1	Q9M0V0
			AtmFDX2	Q8S904
			AtmFDXlike	Q9SRR8
	Chlorophyta	*Chlamydomonas rheinhardtii*	CrcFDX	Q2HZ24
			CrFDX1	A8JHR1
			CrNUOP3	Q6QAY1
	Rhodophyta	*Cyanidioschyzon merolae*	CmcFDX1	M1V5H0
			CmcFDX2	Q85FT5
			CmcFDX2	Q85FT5
Cryptista	Cryptophyta	*Guillardia theta*	GtcFDX	O78510
			GtFDX1	L1IPS0
			GtFDX2	L1J5A6
	Picozoa	*Picomonas judeaskeda*	no ferredoxins identified	
	Malawimonadea	*Malawimonas jakobiformis*	no ferredoxins identified	
Haptista	Haptophyta	*Emiliania huxleyi*	EhcFDX	R1DCW6
			EhFDX1	R1DSP5
			EhFDX2	R1D133
Stramenopila	Eustigmatophycea	*Nannochloropsis gaditana*	NgcFDX1	W7U5Y2
			NgcFDX2	W7TUL4
			NgFDX1	W7TQA0
			NgFDX2	K8YUL7
			NgFDX3	W7TRL6
	Diatoms	*Thalassiosira oceanica*	TocFDX	K0RP06
			ToFDX1	K0TE65
			ToFDX2	K0SI29
			ToFDX3	K0RID3
	Oomyceta	*Phytophthora infestans*	PiPDX1	D0MSF6
			PiPFD2	A0A833RND4

Group	Class	Organism	Name	Uniprot
Alveolata	Apicomplexa	*Toxoplasma gondii*	TgFDX1	B6KFF5
			TgFDX2	B9Q746
	Ciliophora	*Tetrahymena thermophila*	TtFDX1	Q22VV0
			TtFDX2	I7M2B0
			TtNDUFX	Q22W11
Rhizaria	Foraminifera	*Reticulomyxa filosa*	RfFDX1	X6PDJ4
			RfFDX2	X6NJJ3
Discoba	Kinetoplastida	*Trypanosoma brucei*	TbFDX1	Q57VT5
			TbFDX2	Q584K7
	Euglenoidea	*Euglena viridis*	EvcFDX	P22341
Metamonada	Diplomonadida	*Giardia intestinalis*	GiFDX	E2RU02
CRuMS	Mantomonada	*Mantomonas plastica*	no ferredoxins identified	

KEELING, P. J. & BURKI, F. 2019. Progress towards the Tree of Eukaryotes. *Curr Biol,* 29, R808-R817.

Supplemental Table S2. Primers used in this study

Primer name	Primer sequence	Use
AtmFDX1for	CCCCCCCCCATATGTCCTCTGAGAATGGTGAT	Cloning in pET vectors
AtmFDX2for	CCCCCCCCCATATGACCTCTGAGAAAGGTGGC	Cloning in pET vectors
AtmFDX1/2rev	CCCCGGATCCCTAATGAGGTTTTGGAACAAACCC	Cloning in pET vectors
AtmFDX-like for	CCCCCCCCATATGGGATCGAAGAAAGTCTCT	Cloning in pET vectors
AtmFDX-like L85C for	CCAGCATCGCATAGAT**GT**GATGACATCGAGGCT	
AtmFDX-like rev	CCCCGGATCCTTACGGAATATCCCAAGG	Cloning in pET vectors
AtmFDX-like L85C rev	AGCCTCGATGTCATC**AC**ATCTATGCGATGCTGG	
SALK015333RP	ATGTCCTGCTTGTATCCATCG	Genotyping SALK_015333
SALK015333LP	ATAGTCCCCGTAATATTGGCG	Genotyping SALK_015333
SALK_LBb1.3	ATTTTGCCGATTTCGGAAC	Genotyping SALK_015333
SAIL434RP	ACACAACTTGGCAACCAAGAC	Genotyping SAIL_434_E06
SAIL434LP	AAACGCGATTTGAACAATACG	Genotyping SAIL_434_E06
SAIL_LB3	TAGCATCTGAATTTCATAACCAATCTCGATACAC	Genotyping SAIL_434_E06
Wisc204_RP	AAGGCTTAGCTTCAGGGACAG	Genotyping WiscDsLoxHs204_01A
Wisc204_LP	AAACGCGATTTGAACAATACG	Genotyping WiscDsLoxHs204_01A
Wisc_LB	TGATCCATGTAGATTTCCCGGACATGAAG	Genotyping WiscDsLoxHs204_01A
oJF212	tgccAAGCTTcgacttgcct	Building of pJF1033
oJF215	attcACTAGTataaccatggtattgg	Building of pJF1033
oJF398	accaGGTCTCaATTGAAACCGGAAAAGTGAGTGAgtt ttagagctagaaatagcaag	Building of pJF1120
oJF399	tggtGGTCTCtAAACCTGCGATCTGAACCTCGCACaa tctcttagtcgactctacc	Building of pJF1120
mFDXI_ScrF	AACAGACCAATGAACGATAGCC	Screening of CRISPR lines
mFDXI_ScrR	CGGAATATCCCAAGGCTTAG	Screening of CRISPR lines
Cas9_F	GTGCCAGCTGCTGACAAGAAG	Detection of Cas9 in the deletion lines
Cas9_R	AGAGCCTAGCGGACAGGATAGC	Detection of Cas9 in the deletion lines
mFDXI_GG_for	GGGGACAAGTTTGTACAAAAAAGCAGGCTTCCCA CAAGAATAGCTCTTGCGC	Cloning in pGWB3 and pGWB4

mFDXI_GG_rev	GGGGACCACTTTGTACAAGAAAGCTGGGTCCGGAATATCCCAAGGCTT	Cloning in pGWB3 and pGWB4
mFDXI_Com_F	GGGGACAAGTTTGTACAAAAAAGCAGGCTTCATGGCGACGACTCTTCAG	Cloning in pGWB2
mFDXI_Com_R	GGGGACCACTTTGTACAAGAAAGCTGGGTCTTACGGAATATCCCAAGGCTT	Cloning in pGWB2
